# Symptomatic Expanding Porencephalic Cyst after Evacuation of Intracerebral Hematoma in an Adult: A Case Report

**DOI:** 10.31662/jmaj.2024-0189

**Published:** 2025-02-07

**Authors:** Yoshiaki Takamura

**Affiliations:** 1 Department of Neurosurgery, Higashiosaka City Medical Center, Osaka, Japan

**Keywords:** porencephalic cyst, intracerebral hematoma, adult case

## Abstract

Porencephalic cysts are very rare in adults. Herein, we present a case of an 88-year-old man with a symptomatic expanding porencephalic cyst after intracerebral hematoma evacuation. He was admitted because of disturbed consciousness and right hemiparesis. A computed tomography (CT) showed a large subcortical hematoma in the left parietal lobe. Hematoma evacuation was performed, his consciousness level improved but gradually deteriorated. Follow-up CT revealed a new cystic lesion with perifocal edema at the hematoma site, with progressive expansion of the cyst. Cyst drainage and -peritoneal shunt placement were performed on postoperative day 14; consequently, his symptoms improved. Considerably, a porencephalic cyst have developed because the cerebrospinal fluid flowed into the closed hematoma cavity from the ventricle owing to the osmotic pressure difference between the ventricle and the hematoma cavity.

## Introduction

Porencephalic cysts are usually congenital and can be seen in neonates. It is rare in adults, with only a few reported cases. Herein, we here present a case of an 88-year-old man with a symptomatic expanding porencephalic cyst after the intracerebral hematoma evacuation.

## Case Report

**Figure 1.**
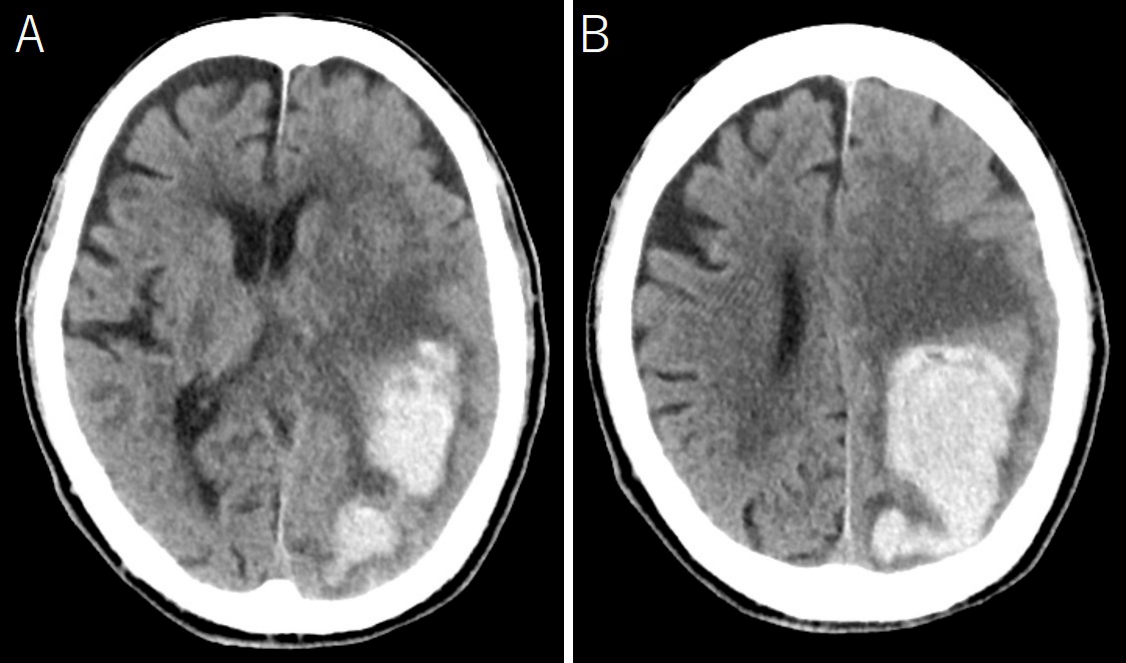
Computed tomography upon admission showed a large subcortical hematoma in the left parietal lobe.

**Figure 2.**
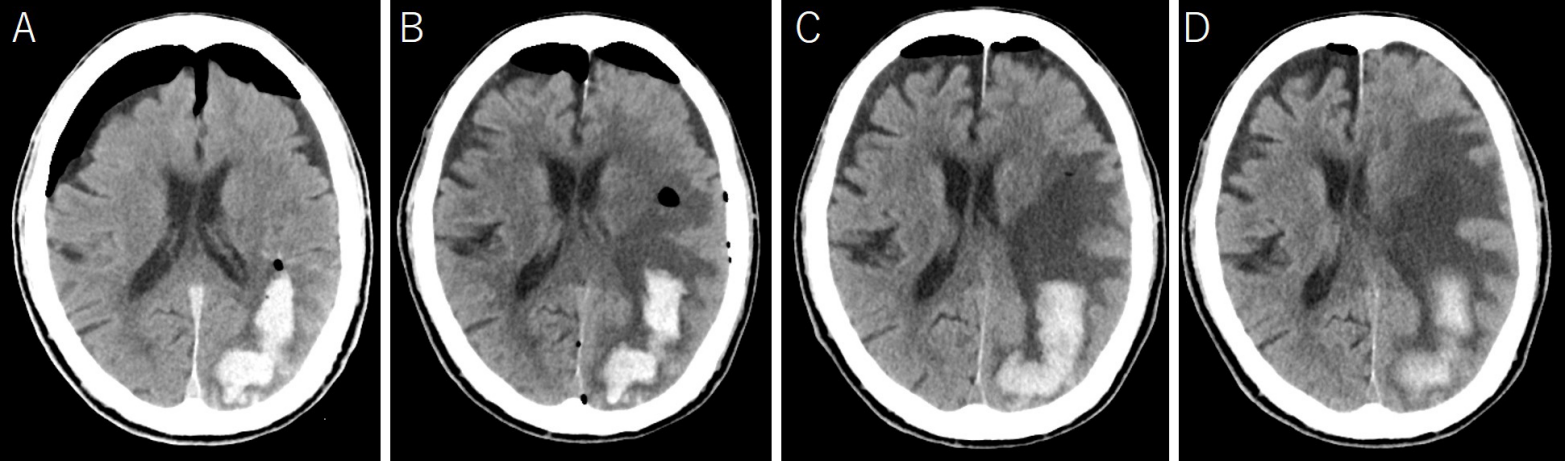
Serial computed tomography (CT) after the first operation revealed the development of a cystic lesion at hematoma and cyst expansion sites. CT immediately after the operation (A); CT on postoperative day 2 (B); CT on postoperative day 8 (C); CT on postoperative day 13 (D).

**Figure 3.**
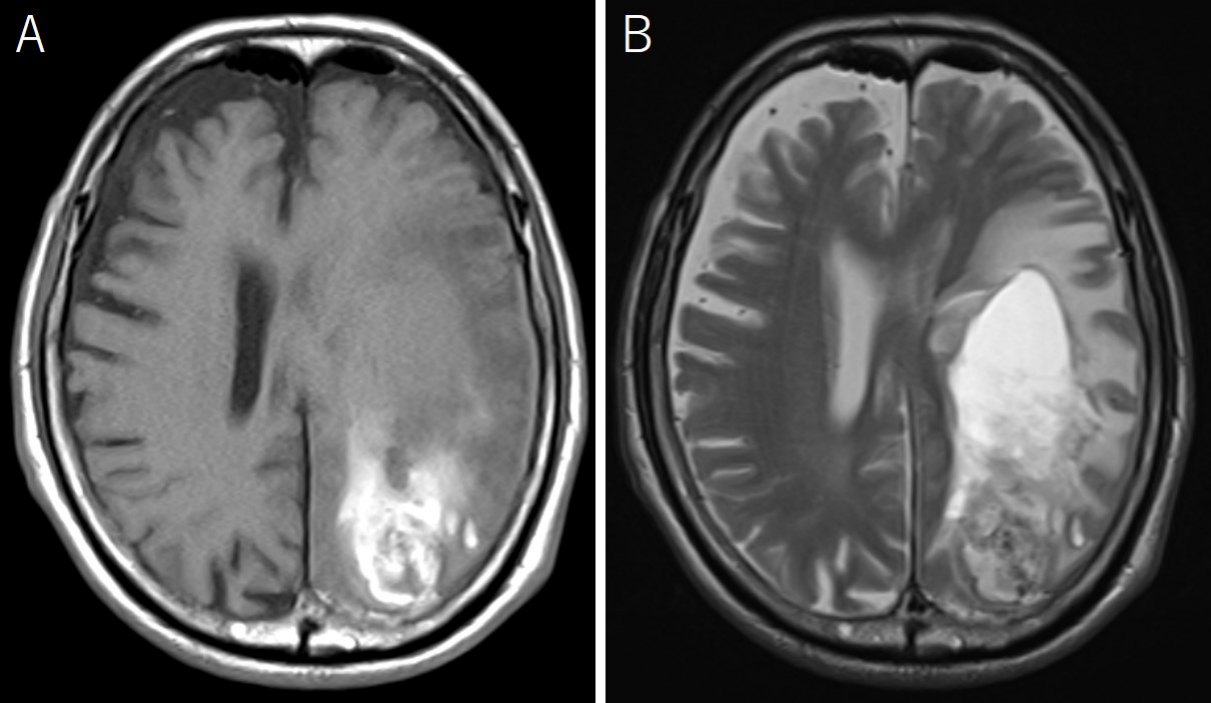
Magnetic resonance images on postoperative day 12; cyst showed iso intensity in the T1-weighted image (A) and marked high intensity in the T2-weighted image (B).

**Figure 4.**
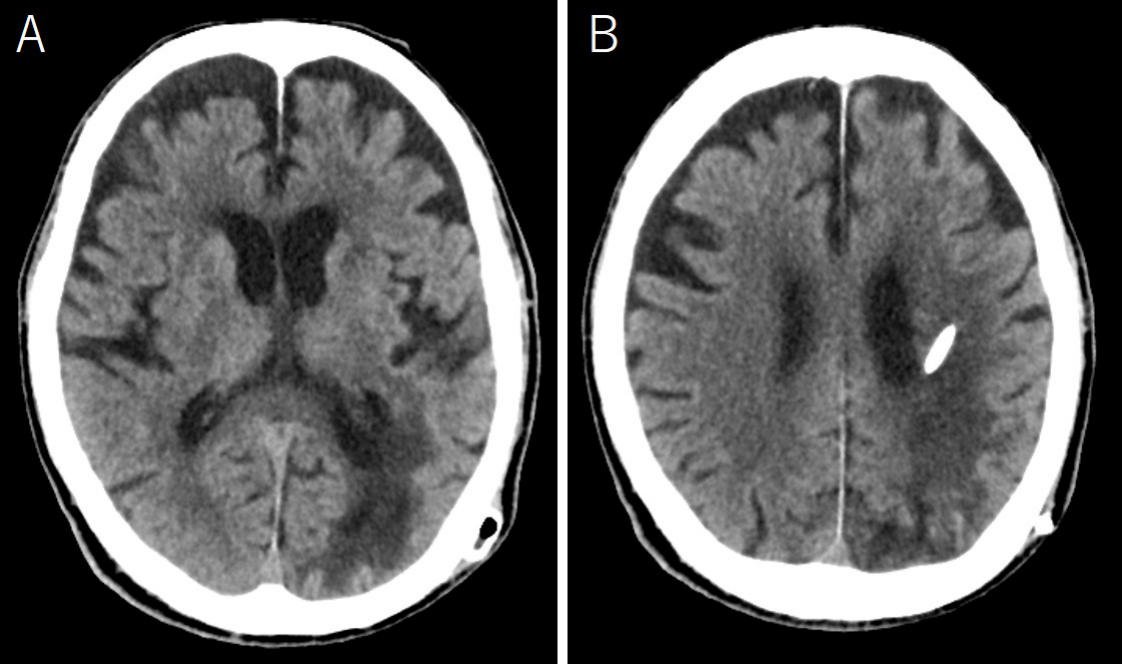
Computed tomography after the second operation revealed cyst shrinkage.

An 88-year-old man was admitted because of disturbed consciousness and right hemiparesis. A computed tomography (CT) scan showed a large subcortical hematoma in the left parietal lobe (Figure 1). Intraventricle hematoma was not observed. Hematoma evacuation was performed with a small craniotomy; the ventricle was not opened. His consciousness level improved but gradually deteriorated. A follow-up CT revealed a new cystic lesion with perifocal edema at the hematoma site, with a progressive expansion of the cyst (Figure 2). Upon magnetic resonance imaging on postoperative day 12, the cyst showed iso and high intensities on T1- and T2-weighted images, respectively (Figure 3). Cyst drainage and -peritoneal shunt placement were performed on postoperative day 14. The cyst fluid specific gravity, white blood cell, and protein level were 1.034, 3,639 cells/μL, and 4,079 mg/dL, respectively. Postoperative CT showed cyst shrinkage (Figure 4), and his symptoms improved.

## Discussion

Porencephalic cyst secondary to intracerebral hemorrhage in adult is extremely rare, with only a few reported cases ^(1), (2), (3)^. Considerably, the cerebrospinal fluid (CSF) in the ventricle flows into the cyst. Courville reported an autopsy case with a posthemorrhagic cyst. They reported that because the cyst was adjacent to the ventricle, the fluid within the cyst was derived from the CSF rather than the surrounding cerebral tissue ^(4)^. Sinagawa et al. reported a case similar to ours, speculating that the cyst expansion could be attributed to a check valve mechanism between the ventricle and the cavity from which the hematoma was removed ^(3)^.

Meanwhile, Yamaguchi et al reported a case of hematoma expansion caused by the trapped CSF in subacute phase intracerebral hemorrhage ^(5)^; in this case, there was a thin lateral ventricle wall with a tiny hole at the bottom of the hematoma cavity. Therefore, the clot covering the tiny hole in the hyperacute and acute phases may melt with time, leading to weakened adhesion and functioning, similar to that of a check valve. The CSF could move into the hematoma cavity from the lateral ventricle. Thin ventricle walls may act as a semipermeable membrane, allowing the fluid to pass. The CSF might move into the hematoma cavity owing to the high osmotic pressure caused by the hematoma ^(2), (5)^.

Herein, because the hematoma cavity was adjacent to the ventricle and the cyst fluid was a hyperosmotic solution, the CSF flowed into the hematoma cavity from the ventricle owing to the osmotic pressure difference between the ventricle and the hematoma cavity. If the hematoma cavity is communicated to the arachnoid space, the cyst may not develop. However, in this case, the hematoma cavity was closed with adhesion; therefore, the cyst developed and expanded.

The functioning of a check valve mechanism is unknown. Reportedly, a porencephalic cyst was treated by cyst drainage after the intracerebral hematoma evacuation and did not recur ^(3)^. Although cyst-peritoneal shunt replacement was performed in this case, the cyst could only be treated by drainage. Considerably, a check valve mechanism only attribute cyst development is unlikely. The osmotic pressure difference was corrected by removing the cyst fluid, which was hyperosmotic solution, and the hematoma cavity was opened by drainage. Considering that cyst drainage is useful, Wynne et al. reported a case of a porencephalic cyst in an adult, treated by endoscopic fenestration of the cyst membrane into the lateral ventricle ^(6)^. Given that the neuroendoscopic surgery is widely used nowadays, the endoscopic fenestration could be an option for the surgical management of porencephalic cyst.

## Article Information

### Conflicts of Interest

None

### Author Contributions

The author contributed to the study conception and design, performed data collection, made substantial contributions to data analyses and interpretation, and wrote this manuscript.

### Approval by Institutional Review Board (IRB)

Not applicable

### Informed Consent

The patient authorized the publication of the case while requesting the confidentiality of his identity.

## References

[1] Cantu RC, LeMay M. Porencephaly caused by intracerebral hemorrhage. Radiology. 1967;88(3):526-30. 5297568 10.1148/88.3.526

[2] Furlow LT, Carr AD, Wattenberg C. Spontaneous cerebral hemorrhage: the surgical treatment of selected cases. Surgery. 1941;9(5):758-70.

[3] Shinagawa H, Matsuo A, Ogawa Y, et al. [Early development of a symptomatic expanding porencephalic cyst in the hematoma cavity after subcortical hematoma removal in an adult: a case report]. No Shinkei Geka. 2020;48(11):1035-42. Japanese. 33199661 10.11477/mf.1436204317

[4] Courville CB. Intracerebral hematoma: its pathology and pathogenesis. Arch Neurol Psychiatry. 1957;77(5):464-72. 13410201

[5] Yamaguchi S, Yoshida M, Iwanaga M. Hematoma expansion caused by trapped cerebrospinal fluid in subacute phase intracerebral hemorrhage: a case report. Surg Neurol Int. 2022;13:86. 35399899 10.25259/SNI_955_2021 PMC8986653

[6] Wynne D, Abdul Jalil MF, Dhillon R. Endoscopic fenestration of a symptomatic porencephalic cyst in an adult. World Neurosurg. 2020;141:245-6. 32569761 10.1016/j.wneu.2020.06.092

